# Correction: An assessment of factors for the cruise port of call selection: The modified fuzzy Analytic Hierarchy Process

**DOI:** 10.1371/journal.pone.0316284

**Published:** 2024-12-19

**Authors:** Thang Quyet Nguyen, Quynh Manh Doan, Lan Thi Tuyet Ngo

The ORCID iD is missing from the first author and the one listed to the second author is incorrect. Please see the authors’ respective ORCID iDs here:

Thang Quyet Nguyen’s ORCID iD is: 0000-0002-7451-902X (https://orcid.org/0000-0002-7451-902X).

Quynh Manh Doan’s ORCID iD is: 0000-0001-6692-3615 (https://orcid.org/0000-0001-6692-3615).

## References

[1] Nguyen TQ, Doan QM, Ngo LTT (2024) An assessment of factors for the cruise port of call selection: The modified fuzzy Analytic Hierarchy Process. PLoS ONE 19(2): e0297293. doi: 10.1371/journal.pone.0297293 38324541 PMC10849245

