# Supplementary material for: An assessment of factors for the cruise port of call selection: The modified fuzzy Analytic Hierarchy Process
Source: PLoS One. 2024 Feb 7;19(2):e0297293. doi: 10.1371/journal.pone.0297293 (PMC10849245; doi:10.1371/journal.pone.0297293)
Supplement: S1 File — (PDF) [file pone.0297293.s001.pdf]

Sample data for respondents' profile

No.	Age	COs or POs	Education	Seniority	Job title
1	41-50	POs	College	10~15	Manager
2	Over 61	COs	Master or above	16-20	Senior staff
3	41-50	COs	College	10~15	Manager
4	41-50	POs	College	10~15	Manager
5	41-50	COs	College	10~15	Manager
6	41-50	COs	College	10~15	Manager
7	Over 61	COs	Master or above	16-20	Senior staff
8	Over 61	COs	Master or above	Over 21	Senior staff
9	41-50	COs	College	10~15	Manager
10	41-50	POs	College	10~15	Manager
11	41-50	COs	College	10~15	Manager
12	Over 61	COs	Master or above	Over 21	Senior staff
13	41-50	COs	College	10~15	Manager
14	51-60	COs	College	10~15	Senior staff
15	41-50	COs	College	10~15	Manager
16	51-60	POs	Master or above	16-20	Senior staff
17	41-50	POs	Master or above	16-20	Senior staff
18	41-50	POs	College	10~15	Manager
19	41-50	POs	College	10~15	Senior staff
20	51-60	COs	College	10~15	Senior staff
21	51-60	POs	College	10~15	Manager
22	41-50	POs	College	10~15	Senior staff
23	41-50	COs	College	10~15	Manager
24	41-50	POs	College	10~15	Manager

Sample data for the dimensions

Respondent 1

Individual reciprocal matrix

	PE	PT	PF	PM
PE	1	1	2	0.333333333
PT	1.0	1	3	1
PF	0.5	0.3	1	0.5
PM	3.0	1.0	2.0	1

Respondent 2

Individual reciprocal matrix

	PE	PT	PF	PM
PE	1	1	0.2	0.2
PT	1.0	1	0.125	0.142857143
PF	5.0	8.0	1	1
PM	5.0	7.0	1.0	1

Respondent 3

Individual reciprocal matrix

	PE	PT	PF	PM
PE	1	0.11111111	1	0.111111111
PT	9.0	1	3	1
PF	1.0	0.3	1	0.333333333
PM	9.0	1.0	3.0	1

Respondent 4

Individual reciprocal matrix

	PE	PT	PF	PM
PE	1	0.125	3	3
PT	8.0	1	3	3
PF	0.3	0.3	1	0.25
PM	0.3	0.3	4.0	1

Respondent 5

Individual reciprocal matrix

	PE	PT	PF	PM
PE	1	0.14285714	1	0.142857143
PT	7.0	1	7	3
PF	1.0	0.1	1	0.166666667
PM	7.0	0.3	6.0	1

Respondent 6

Individual reciprocal matrix

	PE	PT	PF	PM
PE	1	0.11111111	1	0.111111111
PT	9.0	1	9	5
PF	1.0	0.1	1	0.333333333
PM	9.0	0.2	3.0	1

Respondent 7

Individual reciprocal matrix

	PE	PT	PF	PM
PE	1	0.11111111	1	0.25
PT	9.0	1	9	5
PF	1.0	0.1	1	0.125
PM	4.0	0.2	8.0	1

Respondent 8

Individual reciprocal matrix

	PE	PT	PF	PM
PE	1	3	0.2	0.2
PT	0.3	1	0.125	0.142857143
PF	5.0	8.0	1	3
PM	5.0	7.0	0.3	1

Respondent 9

Individual reciprocal matrix

	PE	PT	PF	PM
PE	1	2	0.2	0.333333333
PT	0.5	1	0.125	0.142857143
PF	5.0	8.0	1	3
PM	3.0	7.0	0.3	1

Respondent 10

Individual reciprocal matrix

	PE	PT	PF	PM
PE	1	4	0.16666667	0.333333333
PT	0.3	1	0.125	0.142857143
PF	6.0	8.0	1	2
PM	3.0	7.0	0.5	1

Respondent 11

Individual reciprocal matrix

	PE	PT	PF	PM
PE	1	3	0.2	0.125
PT	0.3	1	0.125	0.142857143
PF	5.0	8.0	1	1
PM	8.0	7.0	1.0	1

Respondent 12

Individual reciprocal matrix

	PE	PT	PF	PM
PE	1	0.11111111	1	0.111111111
PT	9.0	1	3	2
PF	1.0	0.3	1	0.333333333
PM	9.0	0.5	3.0	1

Respondent 13

Individual reciprocal matrix

	PE	PT	PF	PM
PE	1	0.11111111	1	0.25
PT	9.0	1	6	5
PF	1.0	0.2	1	0.125
PM	4.0	0.2	8.0	1

Respondent 14

Individual reciprocal matrix

	PE	PT	PF	PM
PE	1	0.11111111	1	0.5
PT	9.0	1	3	2
PF	1.0	0.3	1	0.333333333
PM	2.0	0.5	3.0	1

Respondent 15

Individual reciprocal matrix

	PE	PT	PF	PM
PE	1	0.11111111	0.14285714	0.5
PT	9.0	1	0.5	3
PF	7.0	2.0	1	8
PM	2.0	0.3	0.1	1

Respondent 16

Individual reciprocal matrix

	PE	PT	PF	PM
PE	1	0.11111111	0.14285714	0.5
PT	9.0	1	0.25	3
PF	7.0	4.0	1	8
PM	2.0	0.3	0.1	1

Respondent 17

Individual reciprocal matrix

	PE	PT	PF	PM
PE	1	0.11111111	1	0.5
PT	9.0	1	3	4
PF	1.0	0.3	1	0.333333333
PM	2.0	0.3	3.0	1

Respondent 18

Individual reciprocal matrix

	PE	PT	PF	PM
PE	1	0.11111111	1	0.25
PT	9.0	1	3	2
PF	1.0	0.3	1	0.333333333
PM	4.0	0.5	3.0	1

Respondent 19

Individual reciprocal matrix

	PE	PT	PF	PM
PE	1	3	3	0.2
PT	0.3	1	0.2	0.2
PF	0.3	5.0	1	0.2
PM	5.0	5.0	5.0	1

Respondent 20

Individual reciprocal matrix

	PE	PT	PF	PM
PE	1	2	3	0.333333333
PT	0.5	1	5	0.142857143
PF	0.3	0.2	1	0.142857143
PM	3.0	7.0	7.0	1

Respondent 21

Individual reciprocal matrix

	PE	PT	PF	PM
PE	1	1	3	0.2
PT	1.0	1	1	0.333333333
PF	0.3	1.0	1	0.2
PM	5.0	3.0	5.0	1

Respondent 22

Individual reciprocal matrix

	PE	PT	PF	PM
PE	1	3	0.333333	1
PT	0.3	1	0.2	0.142857143
PF	3.0	5.0	1	1
PM	1.0	7.0	1.0	1

Respondent 23

Individual reciprocal matrix

	PE	PT	PF	PM
PE	1	1	0.333333	0.2
PT	1.0	1	1	1
PF	3.0	1.0	1	1
PM	5.0	1.0	1.0	1

Respondent 24

Individual reciprocal matrix

	PE	PT	PF	PM
PE	1	5	3	1
PT	0.2	1	0.33333333	0.142857143
PF	0.3	3.0	1	1
PM	1.0	7.0	1.0	1

Sample data for the PE dimension

Respondent 1

Individual reciprocal matrix

	PE1	PE2	PE3	PE4
PE1	1	1	5	0.5
PE2	1.0	1	3	1
PE3	0.2	0.3	1	0.333333333
PE4	2.0	1.0	3.0	1

Respondent 2

Individual reciprocal matrix

	PE1	PE2	PE3	PE4
PE1	1	3	0.2	0.2
PE2	0.3	1	0.33333333	0.142857143
PE3	5.0	3.0	1	1
PE4	5.0	7.0	1.0	1

Respondent 3

Individual reciprocal matrix

	PE1	PE2	PE3	PE4
PE1	1	0.33333333	1	0.333333333
PE2	3.0	1	3	4
PE3	1.0	0.3	1	0.333333333
PE4	3.0	0.3	3.0	1

Respondent 4

Individual reciprocal matrix

	PE1	PE2	PE3	PE4
PE1	1	0.33333333	3	4
PE2	3.0	1	5	4
PE3	0.3	0.2	1	0.333333333
PE4	0.3	0.3	3.0	1

Respondent 5

Individual reciprocal matrix

	PE1	PE2	PE3	PE4
PE1	1	0.14285714	1	0.333333333
PE2	7.0	1	4	3
PE3	1.0	0.3	1	0.333333333
PE4	3.0	0.3	3.0	1

Respondent 6

Individual reciprocal matrix

	PE1	PE2	PE3	PE4
PE1	1	0.33333333	1	0.5
PE2	3.0	1	3	3
PE3	1.0	0.3	1	0.333333333
PE4	2.0	0.3	3.0	1

Respondent 7

Individual reciprocal matrix

	PE1	PE2	PE3	PE4
PE1	1	1	3	3
PE2	1.0	1	2	4
PE3	0.3	0.5	1	5
PE4	0.3	0.3	0.2	1

Respondent 8

Individual reciprocal matrix

	PE1	PE2	PE3	PE4
PE1	1	1	5	5
PE2	1.0	1	7	7
PE3	0.2	0.1	1	3
PE4	0.2	0.1	0.3	1

Respondent 9

Individual reciprocal matrix

	PE1	PE2	PE3	PE4
PE1	1	1	3	2
PE2	1.0	1	3	4
PE3	0.3	0.3	1	3
PE4	0.5	0.3	0.3	1

Respondent 10

Individual reciprocal matrix

	PE1	PE2	PE3	PE4
PE1	1	1	2	3
PE2	1.0	1	5	3
PE3	0.5	0.2	1	3
PE4	0.3	0.3	0.3	1

Respondent 11

Individual reciprocal matrix

	PE1	PE2	PE3	PE4
PE1	1	1	5	5
PE2	1.0	1	7	4
PE3	0.2	0.1	1	3
PE4	0.2	0.3	0.3	1

Respondent 12

Individual reciprocal matrix

	PE1	PE2	PE3	PE4
PE1	1	2	3	3
PE2	0.5	1	5	1
PE3	0.3	0.2	1	0.333333333
PE4	0.3	1.0	3.0	1

Respondent 13

Individual reciprocal matrix

	PE1	PE2	PE3	PE4
PE1	1	2	3	0.333333333
PE2	0.5	1	1	0.333333333
PE3	0.3	1.0	1	0.2
PE4	3.0	3.0	5.0	1

Respondent 14

Individual reciprocal matrix

	PE1	PE2	PE3	PE4
PE1	1	2	3	0.333333333
PE2	0.5	1	2	0.333333333
PE3	0.3	0.5	1	0.2
PE4	3.0	3.0	5.0	1

Respondent 15

Individual reciprocal matrix

	PE1	PE2	PE3	PE4
PE1	1	4	3	0.333333333
PE2	0.3	1	2	0.333333333
PE3	0.3	0.5	1	0.2
PE4	3.0	3.0	5.0	1

Respondent 16

Individual reciprocal matrix

	PE1	PE2	PE3	PE4
PE1	1	2	3	0.333333333
PE2	0.5	1	1	0.5
PE3	0.3	1.0	1	0.2
PE4	3.0	2.0	5.0	1

Respondent 17

Individual reciprocal matrix

	PE1	PE2	PE3	PE4
PE1	1	2	2	4
PE2	0.5	1	1	3
PE3	0.5	1.0	1	3
PE4	0.3	0.3	0.3	1

Respondent 18

Individual reciprocal matrix

	PE1	PE2	PE3	PE4
PE1	1	3	2	6
PE2	0.3	1	2	4
PE3	0.5	0.5	1	3
PE4	0.2	0.3	0.3	1

Respondent 19

Individual reciprocal matrix

	PE1	PE2	PE3	PE4
PE1	1	4	3	3
PE2	0.3	1	2	3
PE3	0.3	0.5	1	2
PE4	0.3	0.3	0.5	1

Respondent 20

Individual reciprocal matrix

	PE1	PE2	PE3	PE4
PE1	1	3	3	0.333333333
PE2	0.3	1	5	0.142857143
PE3	0.3	0.2	1	0.142857143
PE4	3.0	7.0	7.0	1

Respondent 21

Individual reciprocal matrix

	PE1	PE2	PE3	PE4
PE1	1	1	3	0.2
PE2	1.0	1	1	0.333333333
PE3	0.3	1.0	1	0.2
PE4	5.0	3.0	5.0	1

Respondent 22

Individual reciprocal matrix

	PE1	PE2	PE3	PE4
PE1	1	3	0.333333	1
PE2	0.3	1	0.2	0.142857143
PE3	3.0	5.0	1	1
PE4	1.0	7.0	1.0	1

Respondent 23

Individual reciprocal matrix

	PE1	PE2	PE3	PE4
PE1	1	1	0.333333	0.2
PE2	1.0	1	1	1
PE3	3.0	1.0	1	1
PE4	5.0	1.0	1.0	1

Respondent 24

Individual reciprocal matrix

	PE1	PE2	PE3	PE4
PE1	1	5	3	1
PE2	0.2	1	0.33333333	0.142857143
PE3	0.3	3.0	1	1
PE4	1.0	7.0	1.0	1

Sample data for the PT dimension

Respondent 1

Individual reciprocal matrix

	PT1	PT2	PT3	PT4
PT1	1	1	0.14285714	0.2
PT2	1.0	1	0.33333333	0.142857143
PT3	7.0	3.0	1	1
PT4	5.0	7.0	1.0	1

Respondent 2

Individual reciprocal matrix

	PT1	PT2	PT3	PT4
PT1	1	0.33333333	1	0.2
PT2	3.0	1	1	0.2
PT3	1.0	1.0	1	0.333333333
PT4	5.0	5.0	3.0	1

Respondent 3

Individual reciprocal matrix

	PT1	PT2	PT3	PT4
PT1	1	1	1	5
PT2	1.0	1	1	3
PT3	1.0	1.0	1	2
PT4	0.2	0.3	0.5	1

Respondent 4

Individual reciprocal matrix

	PT1	PT2	PT3	PT4
PT1	1	0.33333333	1	0.2
PT2	3.0	1	7	1
PT3	1.0	0.1	1	0.333333333
PT4	5.0	1.0	3.0	1

Respondent 5

Individual reciprocal matrix

	PT1	PT2	PT3	PT4
PT1	1	0.33333333	1	0.2
PT2	3.0	1	3	0.2
PT3	1.0	0.3	1	0.2
PT4	5.0	5.0	5.0	1

Respondent 6

Individual reciprocal matrix

	PT1	PT2	PT3	PT4
PT1	1	1	1	9
PT2	1.0	1	3	9
PT3	1.0	0.3	1	9
PT4	0.1	0.1	0.1	1

Respondent 7

Individual reciprocal matrix

	PT1	PT2	PT3	PT4
PT1	1	1	3	3
PT2	1.0	1	5	1
PT3	0.3	0.2	1	1
PT4	0.3	1.0	1.0	1

Respondent 8

Individual reciprocal matrix

	PT1	PT2	PT3	PT4
PT1	1	3	3	3
PT2	0.3	1	1	3
PT3	0.3	1.0	1	1
PT4	0.3	0.3	1.0	1

Respondent 9

Individual reciprocal matrix

	PT1	PT2	PT3	PT4
PT1	1	1	1	1
PT2	1.0	1	1	0.333333333
PT3	1.0	1.0	1	0.333333333
PT4	1.0	3.0	3.0	1

Respondent 10

Individual reciprocal matrix

	PT1	PT2	PT3	PT4
PT1	1	1	0.33333333	0.333333333
PT2	1.0	1	1	1
PT3	3.0	1.0	1	1
PT4	3.0	1.0	1.0	1

Respondent 11

Individual reciprocal matrix

	PT1	PT2	PT3	PT4
PT1	1	1	5	5
PT2	1.0	1	5	3
PT3	0.2	0.2	1	3
PT4	0.2	0.3	0.3	1

Respondent 12

Individual reciprocal matrix

	PT1	PT2	PT3	PT4
PT1	1	0.33333333	0.33333333	1
PT2	3.0	1	3	3
PT3	3.0	0.3	1	5
PT4	1.0	0.3	0.2	1

Respondent 13

Individual reciprocal matrix

	PT1	PT2	PT3	PT4
PT1	1	0.33333333	5	0.333333333
PT2	3.0	1	5	1
PT3	0.2	0.2	1	0.2
PT4	3.0	1.0	5.0	1

Respondent 14

Individual reciprocal matrix

	PT1	PT2	PT3	PT4
PT1	1	5	5	5
PT2	0.2	1	1	3
PT3	0.2	1.0	1	1
PT4	0.2	0.3	1.0	1

Respondent 15

Individual reciprocal matrix

	PT1	PT2	PT3	PT4
PT1	1	0.2	0.33333333	0.142857143
PT2	5.0	1	1	0.2
PT3	3.0	1.0	1	0.111111111
PT4	7.0	5.0	9.0	1

Respondent 16

Individual reciprocal matrix

	PT1	PT2	PT3	PT4
PT1	1	1	0.33333333	3
PT2	1.0	1	0.2	1
PT3	3.0	5.0	1	3
PT4	0.3	1.0	0.3	1

Respondent 17

Individual reciprocal matrix

	PT1	PT2	PT3	PT4
PT1	1	3	0.33333333	3
PT2	0.3	1	0.2	3
PT3	3.0	5.0	1	7
PT4	0.3	0.3	0.1	1

Respondent 18

Individual reciprocal matrix

	PT1	PT2	PT3	PT4
PT1	1	1	1	3
PT2	1.0	1	3	3
PT3	1.0	0.3	1	1
PT4	0.3	0.3	1.0	1

Respondent 19

Individual reciprocal matrix

	PT1	PT2	PT3	PT4
PT1	1	0.2	1	5
PT2	5.0	1	3	7
PT3	1.0	0.3	1	5
PT4	0.2	0.1	0.2	1

Respondent 20

Individual reciprocal matrix

	PT1	PT2	PT3	PT4
PT1	1	5	5	7
PT2	0.2	1	1	1
PT3	0.2	1.0	1	1
PT4	0.1	1.0	1.0	1

Respondent 21

Individual reciprocal matrix

	PT1	PT2	PT3	PT4
PT1	1	0.33333333	0.2	0.333333333
PT2	3.0	1	0.2	1
PT3	5.0	5.0	1	5
PT4	3.0	1.0	0.2	1

Respondent 22

Individual reciprocal matrix

	PT1	PT2	PT3	PT4
PT1	1	1	0.14285714	0.2
PT2	1.0	1	0.33333333	0.142857143
PT3	7.0	3.0	1	1
PT4	5.0	7.0	1.0	1

Respondent 23

Individual reciprocal matrix

	PT1	PT2	PT3	PT4
PT1	1	5	1	1
PT2	0.2	1	0.142857	0.2
PT3	1.0	7.0	1	1
PT4	1.0	5.0	1.0	1

Respondent 24

Individual reciprocal matrix

	PT1	PT2	PT3	PT4
PT1	1	1	0.2	1
PT2	1.0	1	0.2	1
PT3	5.0	5.0	1	3
PT4	1.0	1.0	0.3	1

Sample data for the PF dimension

Respondent 1

Individual reciprocal matrix

	PF1	PF2	PF3	PF4
PF1	1	0.33333333	1	0.111111111
PF2	3.0	1	1	0.111111111
PF3	1.0	1.0	1	0.111111111
PF4	9.0	9.0	9.0	1

Respondent 2

Individual reciprocal matrix

	PF1	PF2	PF3	PF4
PF1	1	1	1	0.2
PF2	1.0	1	0.333333	0.2
PF3	1.0	3.0	1	0.333333333
PF4	5.0	5.0	3.0	1

Respondent 3

Individual reciprocal matrix

	PF1	PF2	PF3	PF4
PF1	1	3	1	3
PF2	0.3	1	0.2	0.333333333
PF3	1.0	5.0	1	3
PF4	0.3	3.0	0.3	1

Respondent 4

Individual reciprocal matrix

	PF1	PF2	PF3	PF4
PF1	1	3	1	9
PF2	0.3	1	1	9
PF3	1.0	1.0	1	9
PF4	0.1	0.1	0.1	1

Respondent 5

Individual reciprocal matrix

	PF1	PF2	PF3	PF4
PF1	1	0.14285714	0.14285714	0.142857143
PF2	7.0	1	1	1
PF3	7.0	1.0	1	3
PF4	7.0	1.0	0.3	1

Respondent 6

Individual reciprocal matrix

	PF1	PF2	PF3	PF4
PF1	1	3	0.33333333	0.5
PF2	0.3	1	0.14285714	0.333333333
PF3	3.0	7.0	1	4
PF4	2.0	3.0	0.3	1

Respondent 7

Individual reciprocal matrix

	PF1	PF2	PF3	PF4
PF1	1	0.2	1	0.111111111
PF2	5.0	1	3	0.333333333
PF3	1.0	0.3	1	0.25
PF4	9.0	3.0	4.0	1

Respondent 8

Individual reciprocal matrix

	PF1	PF2	PF3	PF4
PF1	1	4	4	4
PF2	0.3	1	2	1
PF3	0.3	0.5	1	2
PF4	0.3	1.0	0.5	1

Respondent 9

Individual reciprocal matrix

	PF1	PF2	PF3	PF4
PF1	1	1	0.2	0.2
PF2	1.0	1	0.33333333	1
PF3	5.0	3.0	1	3
PF4	5.0	1.0	0.3	1

Respondent 10

Individual reciprocal matrix

	PF1	PF2	PF3	PF4
PF1	1	3	1	3
PF2	0.3	1	0.33333333	1
PF3	1.0	3.0	1	1
PF4	0.3	1.0	1.0	1

Respondent 11

Individual reciprocal matrix

	PF1	PF2	PF3	PF4
PF1	1	1	3	4
PF2	1.0	1	4	5
PF3	0.3	0.3	1	3
PF4	0.3	0.2	0.3	1

Respondent 12

Individual reciprocal matrix

	PF1	PF2	PF3	PF4
PF1	1	5	1	5
PF2	0.2	1	0.2	0.2
PF3	1.0	5.0	1	3
PF4	0.2	5.0	0.3	1

Respondent 13

Individual reciprocal matrix

	PF1	PF2	PF3	PF4
PF1	1	1	3	3
PF2	1.0	1	4	3
PF3	0.3	0.3	1	1
PF4	0.3	0.3	1.0	1

Respondent 14

Individual reciprocal matrix

	PF1	PF2	PF3	PF4
PF1	1	5	5	2
PF2	0.2	1	1	1
PF3	0.2	1.0	1	1
PF4	0.5	1.0	1.0	1

Respondent 15

Individual reciprocal matrix

	PF1	PF2	PF3	PF4
PF1	1	1	3	3
PF2	1.0	1	4	3
PF3	0.3	0.3	1	2
PF4	0.3	0.3	0.5	1

Respondent 16

Individual reciprocal matrix

	PF1	PF2	PF3	PF4
PF1	1	2	3	5
PF2	0.5	1	2	1
PF3	0.3	0.5	1	3
PF4	0.2	1.0	0.3	1

Respondent 17

Individual reciprocal matrix

	PF1	PF2	PF3	PF4
PF1	1	9	9	2
PF2	0.1	1	0.16666667	0.125
PF3	0.1	6.0	1	0.5
PF4	0.5	8.0	2.0	1

Respondent 18

Individual reciprocal matrix

	PF1	PF2	PF3	PF4
PF1	1	0.33333333	1	0.2
PF2	3.0	1	1	0.2
PF3	1.0	1.0	1	0.333333333
PF4	5.0	5.0	3.0	1

Respondent 19

Individual reciprocal matrix

	PF1	PF2	PF3	PF4
PF1	1	1	3	2
PF2	1.0	1	1	1
PF3	0.3	1.0	1	2
PF4	0.5	1.0	0.5	1

Respondent 20

Individual reciprocal matrix

	PF1	PF2	PF3	PF4
PF1	1	1	1	2
PF2	1.0	1	1	1
PF3	1.0	1.0	1	1
PF4	0.5	1.0	1.0	1

Respondent 21

Individual reciprocal matrix

	PF1	PF2	PF3	PF4
PF1	1	1	0.2	0.142857143
PF2	1.0	1	0.142857	0.142857143
PF3	5.0	7.0	1	1
PF4	7.0	7.0	1.0	1

Respondent 22

Individual reciprocal matrix

	PF1	PF2	PF3	PF4
PF1	1	1	0.2	1
PF2	1.0	1	0.333333	3
PF3	5.0	3.0	1	3
PF4	1.0	0.3	0.3	1

Respondent 23

Individual reciprocal matrix

	PF1	PF2	PF3	PF4
PF1	1	3	3	3
PF2	0.3	1	1	1
PF3	0.3	1.0	1	3
PF4	0.3	1.0	0.3	1

Respondent 24

Individual reciprocal matrix

	PF1	PF2	PF3	PF4
PF1	1	1	3	3
PF2	1.0	1	3	3
PF3	0.3	0.3	1	4
PF4	0.3	0.3	0.3	1

Sample data for the PM dimension

Respondent 1

Individual reciprocal matrix

	PM1	PM2	PM3	PM4
PM1	1	1	0.2	0.2
PM2	1.0	1	0.142857	0.2
PM3	5.0	7.0	1	0.333333333
PM4	5.0	5.0	3.0	1

Respondent 2

Individual reciprocal matrix

	PM1	PM2	PM3	PM4
PM1	1	3	1	0.333333333
PM2	0.3	1	1	0.142857143
PM3	1.0	1.0	1	0.2
PM4	3.0	7.0	5.0	1

Respondent 3

Individual reciprocal matrix

	PM1	PM2	PM3	PM4
PM1	1	1	3	5
PM2	1.0	1	5	3
PM3	0.3	0.2	1	3
PM4	0.2	0.3	0.3	1

Respondent 4

Individual reciprocal matrix

	PM1	PM2	PM3	PM4
PM1	1	1	0.33333333	0.333333333
PM2	1.0	1	0.33333333	0.2
PM3	3.0	3.0	1	1
PM4	3.0	5.0	1.0	1

Respondent 5

Individual reciprocal matrix

	PM1	PM2	PM3	PM4
PM1	1	0.33333333	0.2	0.2
PM2	3.0	1	0.2	0.333333333
PM3	5.0	5.0	1	1
PM4	5.0	3.0	1.0	1

Respondent 6

Individual reciprocal matrix

	PM1	PM2	PM3	PM4
PM1	1	1	0.333333	0.2
PM2	1.0	1	1	1
PM3	3.0	1.0	1	1
PM4	5.0	1.0	1.0	1

Respondent 7

Individual reciprocal matrix

	PM1	PM2	PM3	PM4
PM1	1	5	3	1
PM2	0.2	1	0.33333333	0.142857143
PM3	0.3	3.0	1	1
PM4	1.0	7.0	1.0	1

Respondent 8

Individual reciprocal matrix

	PM1	PM2	PM3	PM4
PM1	1	1	1	5
PM2	1.0	1	5	7
PM3	1.0	0.2	1	3
PM4	0.2	0.1	0.3	1

Respondent 9

Individual reciprocal matrix

	PM1	PM2	PM3	PM4
PM1	1	5	5	5
PM2	0.2	1	1	3
PM3	0.2	1.0	1	1
PM4	0.2	0.3	1.0	1

Respondent 10

Individual reciprocal matrix

	PM1	PM2	PM3	PM4
PM1	1	1	1	0.333333333
PM2	1.0	1	0.33333333	0.333333333
PM3	1.0	3.0	1	0.333333333
PM4	3.0	3.0	3.0	1

Respondent 11

Individual reciprocal matrix

	PM1	PM2	PM3	PM4
PM1	1	1	0.2	1
PM2	1.0	1	0.333333	3
PM3	5.0	3.0	1	3
PM4	1.0	0.3	0.3	1

Respondent 12

Individual reciprocal matrix

	PM1	PM2	PM3	PM4
PM1	1	1	3	2
PM2	1.0	1	3	4
PM3	0.3	0.3	1	3
PM4	0.5	0.3	0.3	1

Respondent 13

Individual reciprocal matrix

	PM1	PM2	PM3	PM4
PM1	1	5	1	1
PM2	0.2	1	1	0.333333333
PM3	1.0	1.0	1	0.333333333
PM4	1.0	3.0	3.0	1

Respondent 14

Individual reciprocal matrix

	PM1	PM2	PM3	PM4
PM1	1	0.2	0.2	0.333333333
PM2	5.0	1	3	3
PM3	5.0	0.3	1	1
PM4	3.0	0.3	1.0	1

Respondent 15

Individual reciprocal matrix

	PM1	PM2	PM3	PM4
PM1	1	1	0.33333333	3
PM2	1.0	1	0.2	7
PM3	3.0	5.0	1	9
PM4	0.3	0.1	0.1	1

Respondent 16

Individual reciprocal matrix

	PM1	PM2	PM3	PM4
PM1	1	1	0.2	3
PM2	1.0	1	0.2	7
PM3	5.0	5.0	1	9
PM4	0.3	0.1	0.1	1

Respondent 17

Individual reciprocal matrix

	PM1	PM2	PM3	PM4
PM1	1	3	1	0.2
PM2	0.3	1	0.14285714	0.111111111
PM3	1.0	7.0	1	0.2
PM4	5.0	9.0	5.0	1

Respondent 18

Individual reciprocal matrix

	PM1	PM2	PM3	PM4
PM1	1	0.33333333	0.33333333	0.2
PM2	3.0	1	0.2	0.333333333
PM3	3.0	5.0	1	1
PM4	5.0	3.0	1.0	1

Respondent 19

Individual reciprocal matrix

	PM1	PM2	PM3	PM4
PM1	1	1	0.2	0.2
PM2	1.0	1	0.142857	0.2
PM3	5.0	7.0	1	0.333333333
PM4	5.0	5.0	3.0	1

Respondent 20

Individual reciprocal matrix

	PM1	PM2	PM3	PM4
PM1	1	5	5	9
PM2	0.2	1	1	7
PM3	0.2	1.0	1	7
PM4	0.1	0.1	0.1	1

Respondent 21

Individual reciprocal matrix

	PM1	PM2	PM3	PM4
PM1	1	1	0.2	0.125
PM2	1.0	1	0.142857	0.2
PM3	5.0	7.0	1	0.333333333
PM4	8.0	5.0	3.0	1

Respondent 22

Individual reciprocal matrix

	PM1	PM2	PM3	PM4
PM1	1	1	0.333333	0.333333333
PM2	1.0	1	1	1
PM3	3.0	1.0	1	1
PM4	3.0	1.0	1.0	1

Respondent 23

Individual reciprocal matrix

	PM1	PM2	PM3	PM4
PM1	1	1	3	1
PM2	1.0	1	5	3
PM3	0.3	0.2	1	1
PM4	1.0	0.3	1.0	1

Respondent 24

Individual reciprocal matrix

	PM1	PM2	PM3	PM4
PM1	1	0.2	1	5
PM2	5.0	1	3	5
PM3	1.0	0.3	1	3
PM4	0.2	0.2	0.3	1
