# Supplementary material for: An assessment of factors for the cruise port of call selection: The modified fuzzy Analytic Hierarchy Process
Source: PLoS One. 2024 Feb 7;19(2):e0297293. doi: 10.1371/journal.pone.0297293 (PMC10849245; doi:10.1371/journal.pone.0297293)
Supplement: Attachment — Submitted filename: PlosOne.Responses.pdf [file pone.0297293.s003.pdf]

Lan Thi Tuyet Ngo
ngothituyetlan@dntu.edu.vn
November 27, 2023

Dear Editor-in-Chief,

I hope this letter finds you well. I am writing in response to the valuable feedback provided by the reviewers regarding our manuscript titled "An assessment of factors for the cruise port of call selection: The cognitive-gap model based on the modified fuzzy Analytic Hierarchy Process," which was submitted for consideration in Plos One. I would like to express my sincere gratitude to you and the reviewers for taking the time to evaluate our work and for your constructive comments, which have been instrumental in improving the quality of our research.

We have carefully reviewed the reviewers' comments and suggestions, and we have made the necessary revisions to address their concerns. I am pleased to provide a detailed point-by-point response to each of the reviewers' comments as follows:

The research problem is interesting and helpful in assessing selection factors (SFs) for the cruise port operators. The research process is also rigorous. However, there still are some questions need to be clarified before publication. Please consider the comments below:

(1) The definition of gap is not so appropriate that may confuse readers. I suggest the title of the manuscript delete the gap expression.

[Answer]: Thanks so much for the reviewer's comment. We revised the manuscript title to address this suggestion.

(2) To make the paper more readable, the abstract should be presented as: introduction, purpose, method, result, and conclusion.

[Answer]: Thanks so much for the reviewer's comment. We revised the manuscript abstract to address this suggestion.

(3) The introduction should be enriched by some following suggestions: The Introduction must present the motivations of the study from the point of view of literature gaps. At present, the build-up of the motivations, including the contributions of the study, is quite messy. It is difficult to clearly assess the gaps that are advanced in this work. For example, you mentioned the modified fuzzy AHP without reviewing other approaches to tackling the prioritization of crucial factors in cruise port selection. How does the modified fuzzy AHP work in comparison with other MCDM or problem structuring techniques, e.g., DEMATEL, ANP, interpretive structural modelling, fuzzy decision maps, WINGS, entropy, CRITIC, SWARA, among others. There is a whole lot of literature on this topic. The choice of the modified fuzzy AHP must be properly motivated. Thus, I suggest that the introduction section must be written from a general perspective. The message should encompass the need for a better understanding of crucial choice factors in selecting a cruise port based on the literature and the necessary characteristics of the problem that requires the use of an MCDM method, and eventually the AHP.

[Answer]: Thanks so much for the reviewer's comment. We revised the introduction section to address this suggestion.

(4) In the literature review, the manuscript extensively analyses too many previous studies. However, some key articles about port selection are not incorporated. Authors should search relevant articles by keyword: "port selection" to find the key articles. Further, it will be better if references are updated to 2023.

[Answer]: Thanks so much for the reviewer's comment. We revised the literature review section to update the newest research in terms of port selection.

(5) The research framework fails to provide a thorough and robust justification of the proposed "gap model". What is it all about? How does it advance our understanding of the cruise port selection problem? I suggest delete Section 3.1 since it is not necessary in the manuscript.

[Answer]: Thanks so much for the reviewer's comment. We deleted Section 3.1 as suggested.

(6) The visualization Figure 2 for the original and affected weights between CFi and CFj , is a mistake, should be SFi and SFj. In fact, the figure cannot express the whole conception of the interrelation of SFs, and may confuse the readers. The mathematical description is clear enough. Accordingly, I suggest delete it out of the manuscript.

[Answer]: Thanks so much for the reviewer's comment. We deleted Figure 2 as suggested.

(7) It has been argued that the selection of the research sample is of paramount importance. Accordingly, the paper should elucidate how experts are selected for interview.

[Answer]: Thanks so much for the reviewer's comment. We revised the manuscript to elucidate how experts were selected for interview.

(8) The paper mentions the term the "original weight" and the "affected weight". Please explain what they mean and how they are determined in the paper.

[Answer]: Thanks so much for the reviewer's comment. We would like to explain, as follows:

Original weight is initial value assigned to selection factors (SFs), often in the context of a measurement or parameter. This kind of weight is determined by fuzz AHP. Meanwhile, affected weight is defined as the extent to which a criterion (i.e., SF) affects others. In our manuscript, this type of weight is estaimated via a direct-effect matrix.

(9) Discussion is well-written. But, it will be better if authors compare empirical results with what has been done in the relevant literature. By doing so, the difference and similarity can be clearer.

[Answer]: Thanks so much for the reviewer's comment. We revised the discussion section to address this suggestion.

(10) Finally, there are some grammatical errors in the article, so it needs to be proofread by an English native speaker. Further. there are also several inconsistencies of mathematical notations throughout the manuscript.

[Answer]: Thanks so much for the reviewer’s comment. We got our manuscript froopread by an English native speaker. Besides, mathematical notations throughout the manuscript were checked.

I have attached the revised manuscript along with this letter, highlighting all the changes made for your convenience. We hope that these revisions address the reviewers' concerns and meet the high standards of Plos One.

Once again, I would like to express our gratitude for your and the reviewers' time and effort in evaluating our work. We appreciate your consideration of our revised manuscript and look forward to hearing from you regarding its status. If any further revisions or clarifications are required, we are more than willing to make them promptly.

Thank you for your attention to our work, and we hope for a positive outcome in the review process. Please feel free to contact me at ngothituyetlan@dntu.edu.vn if you have any further questions or require additional information.

Sincerely,

Lan Thi Tuyet Ngo, Ms.

Department of Postgraduate Studies, Dong Nai Technology University, Dong Nai, Vietnam.
