# Supplementary material for: An assessment of factors for the cruise port of call selection: The modified fuzzy Analytic Hierarchy Process
Source: PLoS One. 2024 Feb 7;19(2):e0297293. doi: 10.1371/journal.pone.0297293 (PMC10849245; doi:10.1371/journal.pone.0297293)
Supplement: Attachment — Submitted filename: Plos One(1).docx [file pone.0297293.s002.docx]

The research problem is interesting and helpful in assessing selection factors (SFs) for the cruise port operators. The research process is also rigorous. However, there still are some questions need to be clarified before publication. Please consider the comments below:

(1) The definition of gap is not so appropriate that may confuse readers. I suggest the title of the manuscript delete the gap expression.
(2) To make the paper more readable, the abstract should be presented as: introduction, purpose, method, result, and conclusion.
(3) The introduction should be enriched by some following suggestions: The Introduction must present the motivations of the study from the point of view of literature gaps. At present, the build-up of the motivations, including the contributions of the study, is quite messy. It is difficult to clearly assess the gaps that are advanced in this work. For example, you mentioned the modified fuzzy AHP without reviewing other approaches to tackling the prioritization of crucial factors in cruise port selection. How does the modified fuzzy AHP work in comparison with other MCDM or problem structuring techniques, e.g., DEMATEL, ANP, interpretive structural modelling, fuzzy decision maps, WINGS, entropy, CRITIC, SWARA, among others. There is a whole lot of literature on this topic. The choice of the modified fuzzy AHP must be properly motivated. Thus, I suggest that the introduction section must be written from a general perspective. The message should encompass the need for a better understanding of crucial choice factors in selecting a cruise port based on the literature and the necessary characteristics of the problem that requires the use of an MCDM method, and eventually the AHP.
(4) In the literature review, the manuscript extensively analyses too many previous studies. However, some key articles about port selection are not incorporated. Authors should search relevant articles by keyword: “port selection” to find the key articles. Further, it will be better if references are updated to 2023.
(5) The research framework fails to provide a thorough and robust justification of the proposed “gap model”. What is it all about? How does it advance our understanding of the cruise port selection problem? I suggest delete Section 3.1 since it is not necessary in the manuscript.
(6) The visualization Figure 2 for the original and affected weights between CF_i_ and CF_j_ , is a mistake, should be SFi and SFj. In fact, the figure cannot express the whole conception of the interrelation of SFs, and may confuse the readers. The mathematical description is clear enough. Accordingly, I suggest delete it out of the manuscript.
(7) It has been argued that the selection of the research sample is of paramount importance. Accordingly, the paper should elucidate how experts are selected for interview.
(8) The paper mentions the term the "original weight" and the "affected weight". Please explain what they mean and how they are determined in the paper.
(9) Discussion is well-written. But, it will be better if authors compare empirical results with what has been done in the relevant literature. By doing so, the difference and similarity can be clearer.
(10) Finally, there are some grammatical errors in the article, so it needs to be proofread by an English native speaker. Further. there are also several inconsistencies of mathematical notations throughout the manuscript.
